# Design and Implementation of Dongba Character Font Style Transfer Model Based on AFGAN

**DOI:** 10.3390/s24113424

**Published:** 2024-05-26

**Authors:** Congwang Bao, Yuan Li, En Lu

**Affiliations:** 1 School of Mining and Mechanical Engineering, Liupanshui Normal University, Liupanshui 553000, China; cwbao@lpssy.edu.cn; 2 School of Mechatronic Engineering, China University of Mining and Technology, Xuzhou 221116, China; 3 School of Architecture and Design, China University of Mining and Technology, Xuzhou 221116, China; 4 School of Agricultural Engineering, Jiangsu University, Zhenjiang 212013, China; jsluen@163.com

**Keywords:** GAN, attention mechanism, deep learning, font style transfer, Dongba characters

## Abstract

Dongba characters are ancient ideographic scripts with abstract expressions that differ greatly from modern Chinese characters; directly applying existing methods cannot achieve the font style transfer of Dongba characters. This paper proposes an Attention-based Font style transfer Generative Adversarial Network (AFGAN) method. Based on the characteristics of Dongba character images, two core modules are set up in the proposed AFGAN, namely void constraint and font stroke constraint. In addition, in order to enhance the feature learning ability of the network and improve the style transfer effect, the Convolutional Block Attention Module (CBAM) mechanism is added in the down-sampling stage to help the network better adapt to input font images with different styles. The quantitative and qualitative analyses of the generated font and the real font were conducted by consulting with professional artists based on the newly built small seal script, slender gold script, and Dongba character dataset, and the styles of the small seal script and slender gold script were transferred to Dongba characters. The results indicate that the proposed AFGAN method has advantages in evaluation indexes and visual quality compared to existing networks. At the same time, this method can effectively learn the style features of small seal script and slender gold script, and transfer them to Dongba characters, indicating the effectiveness of this method.

## 1. Introduction

**Figure 1:**
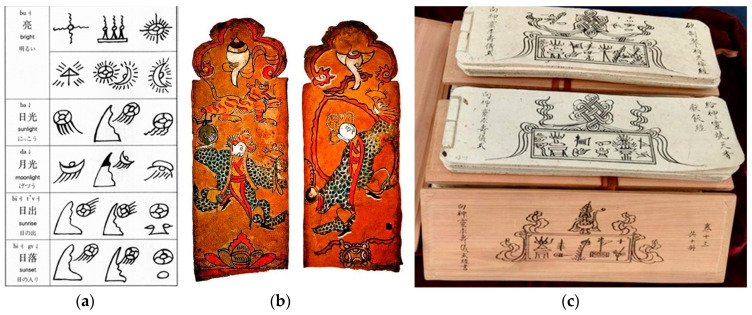
Typical Dongba cultural heritage: (**a**) Dongba characters; (**b**) Dongba paintings; (**c**) Dongba scriptures.

The Naxi minority is one of the few ethnic groups in China that has its own written language and has a long history and unique religious traditions. Dongba (translated as wise man) is a term used by the Naxi minority to refer to traditional religious clergy, who hold multiple positions such as singing, dancing, painting, recording history, and being a doctor. Dongba culture is an ancient culture of the Naxi minority that has been passed down from generation to generation by the Dongba language. It is now mainly composed of Dongba characters, Dongba scriptures, and Dongba paintings, as shown in Figure 1. Considering the Dongba characters, their writing form is more primitive than the inscriptions on bones or tortoise shells of the Shang Dynasty. Due to the fact that there are still inheritors in Yunnan who can read it, it is known as the ‘only living hieroglyphs’ in the world and can be regarded as a shining pearl in the world’s cultural treasure trove [1]. Moreover, the ancient books of Dongba characters have been officially listed in the World Memory List by the United Nations Educational, Scientific and Cultural Organization (UNESCO). However, due to the stagnant development and limited usage of Dongba characters, it cannot be reasonably used in modern life. The Yunnan dialect of Chinese has gradually replaced the Dongba spoken language of the Naxi minority as a tool of communication. Meanwhile, with the development of the times and the influence of modern developed networks, trendy culture, and fast-paced lifestyles, people are unable to calm down and slowly experience these ancient arts. The traditional niche and plain Dongba characters have been gradually forgotten in the corner. These all pose serious challenges to the inheritance of Dongba characters. Therefore, it is urgent to protect, inherit, and develop the Dongba characters of the Naxi minority [2].

Calligraphy is a unique form of written art in China and neighboring countries that has been deeply influenced by Chinese culture. It is known as wordless poetry, intangible dance, pictureless painting, and silent music [3]. Starting from inscriptions on bones or tortoise shells and drum inscriptions, Chinese calligraphy has gradually evolved into five main font styles, including seal script, official script, cursive script, regular script, and running script. They have a unique artistic beauty in terms of writing style, structure, and organization. Excellent calligraphy is not only an artistic expression of language, but also a manifestation of visual thinking. If the unique calligraphy effect of Chinese characters can be integrated into Dongba characters, new forms and application methods of Dongba characters can be innovated, which has important practical significance for the inheritance and protection of Dongba characters [4]. According to statistics, there are about 1500 existing Dongba characters, and the calligraphy design of Dongba characters requires professionals with design knowledge to design each stroke through handwriting, knife carving, and rubbing methods, making this a very challenging task. Meanwhile, compared to modern standard font design, the design of Dongba characters based on ancient Chinese calligraphy is more difficult, requiring designers to proficiently master the standardized writing methods of various ancient Chinese calligraphy fonts, which is difficult to achieve at this stage. Font style transfer is a prominent research direction in the field of computer vision, which involves applying the style of one font to another to generate visually appealing text effects. Therefore, we can use font style transfer technology to transfer Chinese calligraphy to Dongba characters.

In the early research on font style transfer, the traditional method was a Chinese character style transfer model based on statistical calculation models [5], and the simulation of visual thinking was introduced into Chinese character generation [6]. Orbay et al. proposed a font generation method that manually annotated the size and position of partial structures of Chinese characters, extracting the required structures from the input Chinese characters to generate the required Chinese characters [7]. These methods mainly relied on the rules and features of manual design, usually requiring the intervention of professional designers and a lot of manual work. Therefore, it cannot adapt to diverse fonts and is difficult to achieve automation, which limits its practical application.

In recent years, with the development of deep learning, font style transfer algorithms have undergone a revolutionary change. Among them, the most widely used font style transfer network structure is based on the Generative Adversarial Network (GAN) model [8,9]. However, a single GAN model can lead to uncertainty in the mapping combination of the source and target domains, resulting in only one image in the target domain being associated with the source domain, meaning that the generated image will always be a certain image in the same style dataset. The CycleGAN model inherits the idea of the GAN’s adversarial training, which is a framework that can perform image translation using unpaired training data [10]. However, the CycleGAN utilizes the texture features of the underlying images to transfer two different styles of images, which ignores the semantic information of the images, resulting in the CycleGAN not having a good effect in font style transfer. He et al. proposed the ChipGAN to consider the semantic information of images, an end-to-end GAN-based architecture for photo-to-Chinese ink wash painting style transfer [11]. Due to the similarity between ink wash painting and calligraphy font strokes, this model has a great reference value for the style transfer of calligraphy fonts. However, the ChipGAN focuses more on local information during training and does not combine local features to compare with global features, resulting in the loss of some semantic information during style transfer. To this end, we need to improve it.

At present, relevant scholarly research on font style transfer mainly focuses on the artistic transformation of Chinese characters and the generation of calligraphy fonts [3,12,13,14,15,16,17,18], the font style transfer of Mongolian [19], the font style conversion of English and Chinese [20], and the generation of handwritten Western Xia characters [21]. However, there is currently no research on the font style transfer of Dongba characters. In addition, Dongba characters are ancient ideographic scripts with abstract expressions that differ greatly from modern Chinese characters, making it difficult to achieve the font style transfer of Dongba characters. Therefore, it is still a challenge to directly map the calligraphy style of the target Chinese characters onto the Dongba characters while retaining the structural features of the Dongba characters. Based on this, we propose the Attention-based Font style transfer Generative Adversarial Network (AFGAN) algorithm. It starts from two aspects of feature extraction and feature fusion for font style transfer, integrating more features of the font to be learned to reduce the impact of noise on the model, thereby improving the quality and accuracy of Dongba character font style transfer.

The paper is organized as follows: The existing work related to the topic of this paper is introduced in Section 2. In Section 3, the proposed AFGAN model is demonstrated. Section 4 presents the experimental results of the proposed AFGAN model. Section 5 gives brief conclusions and points towards directions for future work.

## 2. Related Works

Font generation (style transfer) is a long-term challenge that many scholars are attempting to solve. In this section, we review some research papers closely related to our work.

### 2.1. Image Style Transfer

Image style transfer refers to extracting styles from one or more images and applying them to another image. In recent years, with the development of deep learning algorithms, supervised learning algorithms based on Convolutional Neural Networks (CNNs) have been widely used in image style transfer. However, the limited scale of manually created paired training image libraries results in poor image generation performance [22]. It was not until Goodfellow et al. proposed the GAN that this field gained new vitality [8]. The typical GAN model consists of two modules: a generator and a discriminator. The generator generates increasingly realistic samples in an attempt to deceive the discriminator, while the discriminator strives to improve its accuracy to distinguish the generated samples from the real samples. As the training progresses, the generator and discriminator continuously iterate and update, ultimately achieving dynamic balance. Conditional GANs (CGANs) introduce image-to-image translations and structured loss functions [23], enabling the network to not only learn the mapping from input image to output image, but also train the loss function of the mapping. This feature makes CGANs suitable for solving image generation problems. Zhu et al. proposed the CycleGAN [10], which solves the problem of mismatched image datasets in many previous networks. However, the CycleGAN requires the training of two GAN models simultaneously, which usually results in slow convergence and a time-consuming training process. Chen et al. proposed the CartoonGAN [24], which greatly reduces the requirements for dataset training and effectively utilizes the performance of GANs. However, the CartoonGAN may encounter serious ambiguous color blocks during the transfer of portrait parts, resulting in unsatisfactory image transfer results. He et al. proposed the ChipGAN, the first weakly supervised deep network architecture to perform photo to Chinese ink wash painting style transfer [11]. Combining the coloring method used in the CGAN, Chen et al. proposed the AnimeGAN, which eliminates the important issue of ambiguous color blocks during the portrait transfer period [25]. However, this method loses numerous facial details. In recent years, more GAN variant models have been proposed, such as the GRA-GAN [26], TwinGAN [27], and FadGAN [28], which have been well-applied in image style transfer.

### 2.2. Font Style Transfer

Font style transfer is a technique for converting font styles, which involves making corresponding changes to the font shape of characters while maintaining the semantic content. Huang et al. proposed a novel CNN model for the classification of Chinese historical calligraphy styles in regular script font, which can classify calligraphy styles and shows superior performance over existing networks [12]. Lyu et al. proposed a deep neural network model that can directly generate calligraphy images from standard font images of Chinese characters [13]. Li et al. applied a CNN model to Mongolian font style transfer [19]. Zhu et al. proposed a deep deformable style transfer network for artistic font style transfer, which can adjust the degree of font deformation according to the style and realize the multiscale artistic style transfer of text [14]. Chang et al. proposed the DenseNet CycleGAN to solve the problem of font mapping from printed fonts to personalized handwriting styles [15]. Zhang et al. improved the generator in the CycleGAN by combining U-NET and ResNet, which can effectively preserve the feature information of the source font during font style transfer [3]. Chen et al. proposed a one-to-many font style transfer model based on the improved StarGAN, but the effect of the font transfer was weakened, and the expression of style features was not sufficient [16]. Sun et al. proposed a novel framework named the Style-Aware Variational Auto-Encoder (SA-VAE) to flexibly generate Chinese characters [17]. Although this method had a certain generalization ability with few samples, the generated images exhibited blurry phenomena. In order to overcome the phenomenon of artifacts and blurring at the bends of the strokes in the generation process of Cuan fonts, Yao et al. proposed a style transfer model of Cuan fonts based on a dense adaptive generation adversarial network [18]. Li et al. developed a novel FTransGAN and applied an end-to-end solution to cross-language font style transfer for the first time [20].

### 2.3. Attention Mechanism

The attention mechanism was first proposed in the field of natural language processing to mitigate the damage caused by a fixed-length vector in the encoder–decoder architecture [20]. Later, Xu et al. applied attention networks to the field of computer vision to solve the problem of image captioning [29]. Yu et al. improved the attention network to a multi-level architecture, obtaining spatial and semantic information from a single image [30]. Chen et al. used additional attention networks to generate attention maps in order to focus more attention on the objects of interest [31]. Yang et al. proposed to add an attention module to predict an attention map to guide the image translation process [32]. Hou et al. proposed an attention-guided single-image translation method using a multi-scale pyramid architecture. It solved the problem of poor image quality in current single-image translation [33]. Tang et al. proposed a new attention-guided GAN (AttentionGAN) for the unpaired image-to-image translation task [34]. Yadav et al. designed a new attention-guided cyclic GAN for thermal-to-visible face transformation (TVA-GAN) by integrating a new attention network [35]. Among them, attention-guided and recursive blocks with initiation modules are used to simplify the learning space and obtain the optimal solution.

## 3. Materials and Methods

### 3.1. Structure of AFGAN

**Figure 2:**
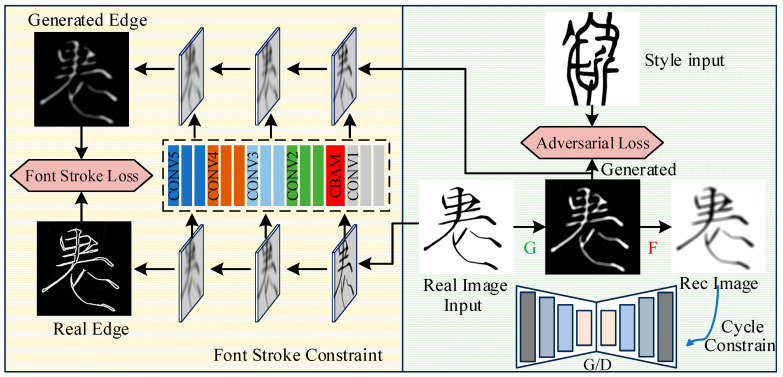
Pipeline of the proposed AFGAN.

Reference [11] proposed the ChipGAN, an end-to-end GAN-based architecture for photo-to-Chinese ink wash painting style transfer. However, the ChipGAN focuses more on local information during training and does not compare local and global features, resulting in the loss of some semantic information during style transfer. Due to the similarity in stroke and other characteristics between Dongba characters and Chinese ink wash painting, the model in Ref. [11] has great reference value for Dongba character font style transfer. Based on this, we propose the AFGAN algorithm, and the pipeline of the proposed AFGAN is illustrated in Figure 2. Based on the characteristics of Dongba character images, this paper proposes two special constraints, namely void constraint and font stroke constraint.

The purpose of void constraint is to generate more realistic results by converting information into imperceptible signals, leaving behind white areas. Given unpaired training sets that are considered as two domains *X* and *Y*, the model includes two mappings: *G*: *X*→*Y* and *F*: *Y*→*X*. According to *G*: *X*→*Y* and its discriminator *D_Y_* [15], the antagonistic loss function is defined as follows:

(1) $$L_{GAN}\left(G,D_{Y},X,Y\right)=E_{y\sim P_{\mathrm{data}\;}(y)}\left[\log D_{Y}(y)\right]+E_{x\sim P_{\mathrm{data}\;}(x)}\left[\log\left(1-D_{Y}(G(x))\right)\right]$$

where *G* attempts to generate samples similar to real samples from domain *Y*, while *D_Y_* attempts to distinguish between fake samples and real samples, and *x* (*x*∈*X*) and *y* (*y*∈*Y*) represent content font image and style font image, respectively.

In addition, a cycle consistency constraint was added to the model [10]. By translating the given font image from domain *X* to target domain *Y* and then back to domain *X*, this produces the same image, i.e., *F*(*G*(*x*)) ≈ *x*. Due to the cycle consistency constraint that requires restoration in both directions, there is also a cycle consistency constraint for each font image y in domain *Y*, i.e., *G*(*F*(*y*)) ≈ *y*. Therefore, the cycle consistency loss function is defined as follows:

(2) $$L_{cyc}(G,F,X,Y)=E_{x\sim P_{\mathrm{data}\;}(x)}\left[\|F(G(x))-x{\|}_{1}\right]+E_{y\sim P_{\mathrm{data}\;}(y)}\left[\|G(F(y))-y{\|}_{1}\right]$$

The above void constraint allows the generated font images to retain some information from the source domain, allowing for the conversion of the generated font images back to the source domain.

Secondly, we developed a font stroke constraint to enhance the consistency between the different level edge maps of the target font images and the generated font images. In order to depict the outline of the font more clearly, the holistically nested edge detector was used to extract all the levels of edges from the input font images. Unlike the problem of regarding the edge detection tasks as a pixel-level binary classification, a multi-level edge detector was trained from a regression perspective to obtain smooth font strokes of different thicknesses [11]. Then, the loss function of edge detection is defined as follows:

(3) $$L_{fontstroke}(G,X)=E_{x\sim P_{\mathrm{data}\;}(x)}\left[-\frac{1}{N}\sum_{i=1}^{N}\mu E{\left(x\right)}_{i}\log E{\left(G(x)\right)}_{i}+(1-\mu )(1-E{\left(x\right)}_{i})+\log(1-E{\left(G(x)\right)}_{i})\right]$$

where parameter *N* is the total number of edge maps of the font images and *μ* is a balancing weight. *μ* = *N*−/*N* and 1 – *μ* = *N*+/*N*. *N−* and *N*+ are the sum of the non-edge and edge probabilities of every pixel in *E*(*x*), respectively.

So, the complete loss function is a linear combination of the above loss functions, and it can be expressed as follows:

(4) $$L\left(G,F,D_{X},D_{Y}\right)=L_{GAN}\left(G,D_{Y},X,Y\right)+L_{GAN}\left(F,D_{X},Y,X\right)+\lambda L_{cyc}(G,F,X,Y)+\gamma L_{fontstroke}(G,X)$$

where parameters *λ* and *γ* control the contributions of the individual loss functions.

At the same time, in order to enhance the feature learning ability of the network, improve the style transfer effect, and enhance the generalization performance, the Convolutional Block Attention Module (CBAM) mechanism was added in the down-sampling stage to help the network better adapt to different input font images. This enabled the proposed method to have more stable and excellent performance in handling any font style that requires transfer learning.

### 3.2. CBAM Mechanism

For the transfer learning of Dongba character art fonts, each neuron does not need to fully understand all the font images, they are only required to understand local information and obtain global information through comprehensive local information. The CBAM extracts informative features by blending cross-channel and spatial information together. It can effectively link local information and global information in convolutional systems, thereby enabling a more effective transmission of information between different branches [34]. Therefore, we introduced the CBAM to extract information from font images at the beginning and provide it to the next stage of the network.

**Figure 3:**
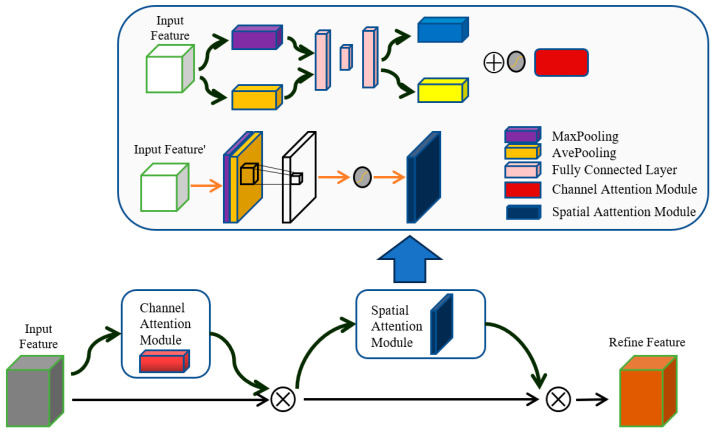
Structure diagram of CBAM mechanism.

In the proposed AFGAN model, the datasets of Dongba characters and small seal script (or slender gold script) are respectively inputted into the GAN, and through the down-sampling structure of convolutional layers, the primary features of font images can be obtained. Then, these features are fed into the CBAM, and the CBAM is divided into two parts: the channel attention part and spatial attention part [33], as shown in Figure 3.

The channel attention module is mainly based on the channel dimension of the feature images for attention weighting. It can adaptively learn the important parts in each channel, thereby enhancing the attention to important information in the font images. In order to better focus on channel attention, we squeezed the spatial dimension of the input feature map. By using average pooling and maximum pooling operations to aggregate the spatial information of the feature map [36], two different spatial context descriptors are generated: $F_{avg}^{C}$ and $F_{max}^{C}$, which represent average pooling features and maximum pooling features, respectively. Then, forward these two descriptors to the shared network to generate our channel attention map $M_{c}\in R^{C\times 1\times 1}$. The shared network is composed of a multi-layer perceptron (MLP) with one hidden layer. When these features are applied to each channel, the final feature vector is obtained by summing them element by element. The corresponding calculation equation is shown as follows [37]:

(5) $$M_{C}(F)=\sigma (MLP(AvgPool(F))+MLP(MaxPool(F)))\;=\sigma (W_{1}(W_{0}(F_{avg}^{C}))+W_{1}(W_{0}(F_{max}^{C})))$$

where *σ* denotes the sigmoid function, $W_{0}\in R^{C/r\times C}$ and $W_{1}\in R^{C\times C/r}$. Note that the MLP weights, *W*_0_ and *W*_1_, are shared for both inputs, and the ReLU activation function is followed by *W*_0_.

The spatial attention module is mainly based on the spatial dimension of feature maps for attention weighting. It can adaptively learn the important parts of each spatial position, thereby enhancing the attention to important areas in the font images. By using average pooling and maximum pooling operations along the channel axis, we concatenated them to generate an efficient feature descriptor [38]. Then, we applied a convolution layer on the connected feature descriptors to generate a spatial attention map $M_{c}(F)\in R^{H\times W}$. In order to aggregate the channel information of the feature map, two 2D maps ($F_{avg}^{S}\in R^{1\times H\times W}$ and $F_{max}^{S}\in R^{1\times H\times W}$) are generated using two pooling operations. Then, a standard convolution layer is used to connect them to generate a 2D spatial attention map. The corresponding calculation equation is shown as follows [37]:

(6) $$M_{S}(F)=\sigma \left(f^{7\times 7}[AvgPool(F);MaxPool(F)]\right)=\sigma (f^{7\times 7}([F_{avg}^{S};F_{max}^{S}]))$$

## 4. Experiment and Result Analysis

### 4.1. Experimental Dataset

**Figure 4:**
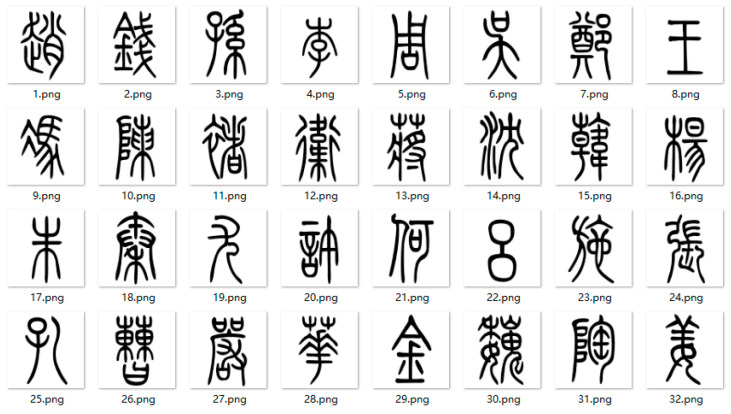
Partial small seal script font dataset.

**Figure 5:**
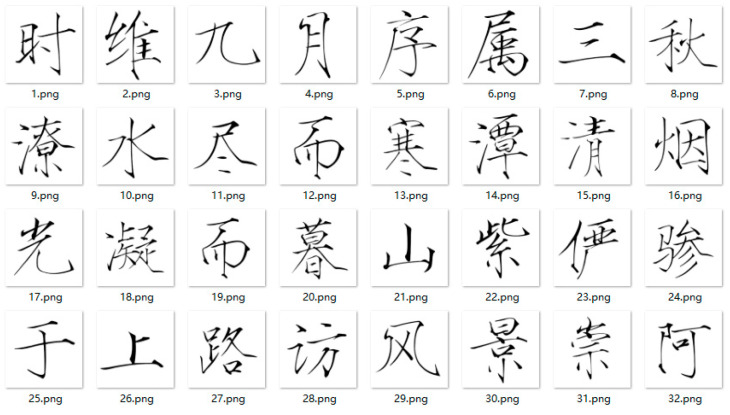
Partial slender gold script font dataset.

There have been many famous calligraphy fonts in Chinese history, such as small seal script, cursive script, regular script, running script, slender gold script, etc. Small seal script was adopted during the Qin Dynasty for the purpose of standardizing the script, and it is the earliest standardized Chinese character set. Slender gold script originated from Emperor Huizong (Ji Zhao) of the Song Dynasty and is characterized by slim yet sturdy strokes. There are significant differences between small seal script and slender gold script in terms of calligraphy style, structure, and other aspects. Therefore, in order to test the performance of the proposed AFGAN in the font style transfer of Dongba characters, these two fonts were selected to establish the source experimental dataset. First, the font packages of small seal script and slender gold script were added to Microsoft Word (ver 2016), and the ‘Hundred Family Surnames’ of small seal script and ‘Preface to Tengwang Pavilion’ of slender gold script were generated based on this, respectively. Subsequently, the Chinese characters in the corresponding small seal script document and slender gold script document were divided into separate image files using Photoshop (ver 2022) software. The number of datasets for the small seal script and slender gold script was 500, and the size of each image was 90.31 mm × 90.31 mm with a resolution of 72. Some experimental datasets are shown in Figure 4 and Figure 5, respectively.

**Figure 6:**
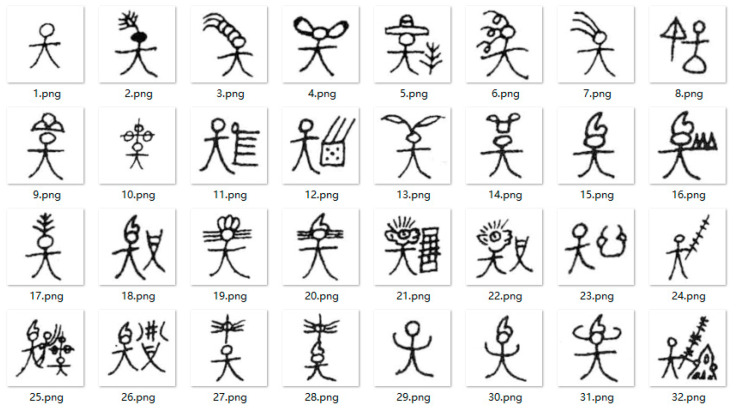
Dongba character font dataset.

The Dongba characters used for style transfer were extracted from Zhao Jingxiu’s “Translations of everyday words for pictographs of Dongba religion (revised edition)” [39]. Similarly, the Dongba characters were divided into separate image files using Photoshop software. The number of datasets for the Dongba characters was 500, and the size of each image was 90.31 × 90.31 mm with a resolution of 72, shown in Figure 6.

### 4.2. Experimental Environment and Parameter Settings

The deep learning tool used in the experiments was Pytorch, and the programming language was Python 3.7. The program was run with the installation of deep learning framework drivers CUDA 8.0 and CUDNN 6.0. The main environment configuration and version information are shown in Table 1.

Selecting ADAM as the optimizer, with an initial learning rate of 10^−3^, the weight decay was set to 10^−6^. At the same time, the learning speed will decrease by half every 50 k iterations, and the number of training rounds is 200.

### 4.3. Result Analysis

To verify the effectiveness of the AFGAN method proposed in this paper, we used the dataset created in Section 4.1 to train all the data to convergence and then compared them with the CycleGAN [10], ChipGAN [11], and AttentionGAN [34], respectively.

#### 4.3.1. Quantitative Analysis

In order to quantitatively analyze the style transfer effect of Dongba characters, the Structure Similarity (SSIM), Mean Square Error (MSE), and Peak Signal to Noise Ratio (PSNR) were used to evaluate the effectiveness of the proposed AFGAN method. SSIM is an indicator that measures the similarity between two images, including brightness, contrast, and structural comparison. The SSIM value was closer to 1.0, and the generated image was closer to the real image. The mathematical expression is shown as follows [18]:

(7) $$\mathrm{SSIM}\left(I_{\mathrm{output}\;},I_{\mathrm{real}\;}\right)=\frac{\left(2\mu_{I_{\mathrm{oupput}}}\mu_{I_{\mathrm{real}\;}}+c_{1}\right)\left(2\sigma_{I_{\mathrm{output}}I_{\mathrm{real}}}+c_{2}\right)}{\left(\mu_{I_{\mathrm{output}}}^{2}+\mu_{I_{\mathrm{real}\;}}^{2}+c_{1}\right)\left(\sigma_{I_{\mathrm{output}}}^{2}+\sigma_{I_{\mathrm{real}\;}}^{2}+c_{2}\right)}$$

where $\mu_{I_{\mathrm{real}\;}}$ and $\mu_{I_{\mathrm{oupput}}}$ represent the average values of the real image and the generated image, respectively; $\sigma_{I_{\mathrm{real}\;}}^{2}$ and $\sigma_{I_{\mathrm{output}}}^{2}$ represent the variance of the real image and the generated image, respectively; $\sigma_{I_{\mathrm{output}}I_{\mathrm{real}}}$ represents the covariance of the real image and the generated image; *c*_1_ = (*k*_1_*L*)^2^ and *c*_2_ = (*k*_2_*L*)^2^ are constants; and *L* is the dynamic range of the pixel values *k*_1_ = 0.01 and *k*_2_ = 0.03.

MSE is an image difference metric that evaluates the differences between two images by calculating the mean square difference of each pixel. A smaller MSE value indicates high image similarity, but it is very sensitive to brightness differences and may not necessarily reflect human visual perception. The mathematical expression is shown as follows [40]:

(8) $$MSE=\frac{1}{mn}\sum_{i=0}^{m-1}\sum_{j=0}^{n-1}{\left[I_{\mathrm{real}}(i,j)-I_{\mathrm{output}}(i,j)\right]}^{2}$$

A higher PSNR value indicates less distortion between the generated image and the real image. The PSNR calculated based on the MSE is shown as follows:

(9) $$PSNR=10\cdot \log_{10}\left(\frac{MAX_{I}^{2}}{MSE}\right)$$

where $MAX_{I}$ is the maximal pixel value of the image. For example, if each pixel is represented by an 8-bit binary, then the $MAX_{I}$ is 255.

Finally, the comparative experimental results are shown in Table 2 and Table 3, respectively. In order to eliminate the contingency of the model, the 10-time average was taken as the final diagnosis result. It can be seen that the proposed AFGAN method performed best in SSIM, MSE, and PSNR indicators, whether it was small seal script or slender gold script. Compared with the second-ranked ChipGAN method, the proposed AFGAN method improved the SSIM by 26.5% (small seal script) and 15.8% (slender gold script), respectively, reduced the MSE by 4.3% (small seal script) and 5.1% (slender gold script), respectively, and improved the PSNR by 11.5% (small seal script) and 10.9% (slender gold script), respectively. In summary, the proposed AFGAN method with the added attention mechanism on the basis of cycle consistency loss and multi-level edge detection had better performance, and its effectiveness was verified.

#### 4.3.2. Qualitative Analysis

**Figure 7:**
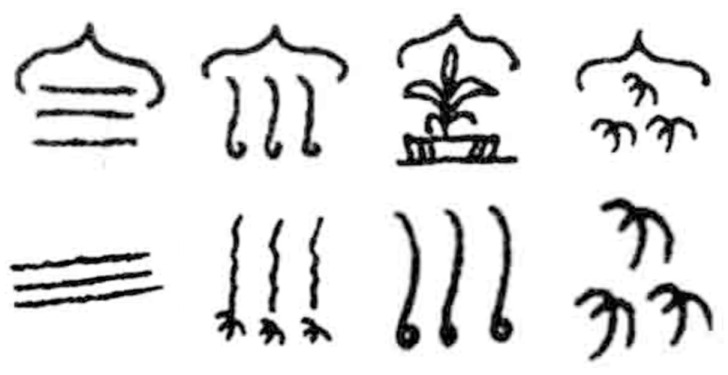
Dongba character test dataset waiting for style transfer. (Their meanings are spring, summer, autumn, winter, wind, frost, rain, and snow, respectively).

**Figure 8:**
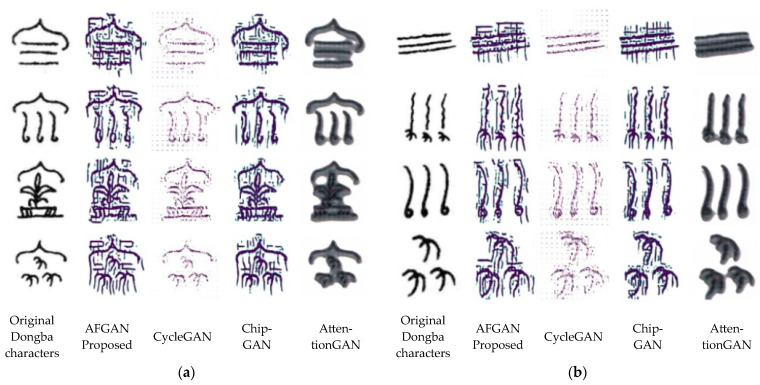
Visual comparison of different methods for transferring Dongba characters to small seal script: (**a**) their meanings are spring, summer, autumn, winter; (**b**) Their meanings are respectively wind, frost, rain, and snow, respectively [10,11,34].

**Figure 9:**
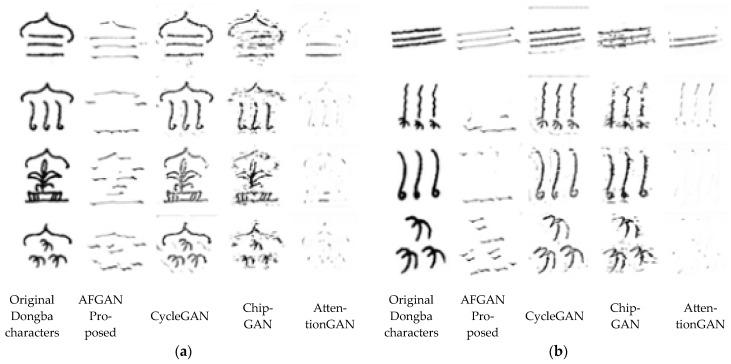
Visual comparison of different methods for transferring Dongba characters to slender gold script: (**a**) their meanings are spring, summer, autumn, winter, respectively; (**b**) their meanings are wind, frost, rain, and snow, respectively [10,11,34].

The Dongba character test dataset for qualitative analysis is shown in Figure 7. The comparative experimental results of different methods (the proposed AFGAN, CycleGAN [10], ChipGAN [11], and AttentionGAN [34]) are shown in Figure 8 and Figure 9, respectively.

Figure 8 shows the visual comparison of different methods for transferring Dongba characters to small seal script. The spatial division of small seal script was balanced and symmetrical, and the strokes of the characters had the characteristics of horizontal and vertical alignment, uniform circular strength, and basic consistency in thickness. Hence, it can be seen that the CycleGAN [10] and AttentionGAN [34] methods did not enable Dongba characters to learn the characteristics of small seal script. The font generated by the ChipGAN [11] method had a certain font style of small seal script. The fonts generated by the proposed AFGAN method exhibited a notably typical small seal font style. Despite minor interference in the generated fonts, which could potentially compromise the readability of Dongba fonts, the overarching aim of this investigation was to achieve an efficient style transformation of Dongba fonts. Upon comparison with alternative methods, the research findings indicated that the proposed method demonstrated a superior font style transfer effect and achieved the expected purpose.

Figure 9 shows the visual comparison of different methods for transferring Dongba characters to slender gold script. The writing style of slender gold script is agile and fast, and its handwriting is slender and vigorous, like gold filament, twisted and turned. Hence, it can be seen that the CycleGAN [10] and AttentionGAN [34] methods did not enable Dongba characters to learn the characteristics of slender gold script. The font generated by the ChipGAN [11] method had a certain font style of slender gold script. The font generated by the proposed AFGAN method had a more typical font style of slender gold script, but the information of the characters themselves was lost.

In conclusion, compared with traditional methods (the CycleGAN [10], ChipGAN [11], and AttentionGAN [34]), the proposed AFGAN method with the added CBAM mechanism on the basis of cycle consistency loss and multi-level edge detection could adjust the details of the generated images based on the input font images, thereby providing more realistic and diverse transfer learning. Meanwhile, the above analyses demonstrate that the proposed AFGAN method can effectively learn the style features of small seal script and slender gold script and transfer them to Dongba characters, indicating the effectiveness of this method.

## 5. Conclusions

This paper proposes an AFGAN method for transferring Chinese calligraphy fonts to Dongba characters. This network included two constraints, void and font strokes, and integrated the CBAM mechanism to better capture key features in the input calligraphy font images. Finally, the experiments were conducted on the newly built small seal script, slender gold script, and Dongba character dataset.

The quantitative analysis results indicated that the proposed AFGAN method performed best in all indicators (the SSIM, MSE and PSNR). This proves that compared to traditional methods (the CycleGAN, ChipGAN, and AttentionGAN), the proposed AFGAN method with the added CBAM mechanism on the basis of cycle consistency loss and multi-level edge detection had better performance. The qualitative analysis results indicated that the traditional CycleGAN and AttentionGAN methods could not transfer the font style of the target Chinese characters to Dongba characters, but the ChipGAN method could learn some font styles. Compared with these traditional methods, the proposed AFGAN method could transfer the more typical font style of the target Chinese characters (whether it was small seal script or slender gold script) to Dongba characters. However, in the process of transferring the font style of the slender gold script to Dongba characters, some information of the characters themselves was lost. In summary, the proposed AFGAN method had advantages in evaluation indexes and visual quality compared to existing networks and its effectiveness was verified.

## Figures and Tables

**Table 1:** Experimental environment.

Software/Hardware	Version/Model
CPU	Intel Core i5-12500H (Santa Clara, CA, USA)
RAM	16 G
GPU	Nvidia RTX3050 (Santa Clara, CA, USA)
Python	3.7
Pytorch-GPU	1.7
CUDA	8.0
CUDNN	6.0

**Table 2:** Comparative experimental results for small seal script.

	Indicators	SSIM	MSE	PSNR
Methods				
CycleGAN [10]		0.256 ± 0.012	303.256 ± 7.35	22.875 ± 1.13
ChipGAN [11]		0.389 ± 0.029	220.185 ± 6.09	25.032 ± 1.71
AttentionGAN [34]		0.278 ± 0.098	414.256 ± 9.82	23.863 ± 2.95
Proposed AFGAN		**0.492 ± 0.011**	**210.663 ± 7.33**	**27.921 ± 1.77**

Note: Bold data represent the optimal experimental results.

**Table 3:** Comparative experimental results for slender gold script.

	Indicators	SSIM	MSE	PSNR
Methods				
CycleGAN [10]		0.262 ± 0.019	300.254 ± 8.19	22.081 ± 1.39
ChipGAN [11]		0.385 ± 0.083	221.952 ± 7.83	24.653 ± 1.87
AttentionGAN [34]		0.271 ± 0.035	418.235 ± 9.97	23.901 ± 3.62
Proposed AFGAN		**0.446 ± 0.026**	**210.632 ± 8.05**	**27.332 ± 1.99**

Note: Bold data represent the optimal experimental results.

## Data Availability

The programs and data related to this paper have been uploaded online. If needed, you can obtain them from the following website: https://github.com/jsluen/AFGAN (accessed on 22 May 2024).
